# 1861. Prevalence of COVID-19 within Predominantly Black Church Congregations in Ontario, Canada

**DOI:** 10.1093/ofid/ofad500.1689

**Published:** 2023-11-27

**Authors:** Nicole Wisener, Alice Litosh, Walter Byrne, Matthew Hwang, Kimberly Thompson, Upton D Allen

**Affiliations:** The Hospital for Sick Children, Thornhill, Ontario, Canada; Hospital for Sick Children, Toronto, Ontario, Canada; Hospital for Sick Children, Toronto, Ontario, Canada; Hospital for Sick Children, Toronto, Ontario, Canada; The Hospital for Sick Children, Thornhill, Ontario, Canada; Hospital Sick Children, Toronto, Ontario, Canada

## Abstract

**Background:**

Seroprevalence data provide useful information on COVID-19 burden among various communities by detecting infection in both symptomatic and asymptomatic persons. One challenge is the extent to which potential participants agree to blood testing. To address this, we conducted a community-based survey to assess infection rates within church congregations to understand COVID-19 prevalence in a closed group setting in contrast to a conventional open community setting.

**Methods:**

A cross-sectional survey was conducted within church congregations consisting of mainly Black members from 09/2022 to 05/2023 in Ontario, Canada. Participants completed the survey in person or online. Demographic data and COVID-19-related information, such as polymerase chain reaction (PCR) or rapid antigen positivity and vaccination status, were collected. Survey data were compared with seroprevalence data collected in a related study.

**Results:**

The survey was completed by 434 persons from 5 different congregations. Male to female ratio 1:2.6. Most participants, 319 (73.5%) completed the survey on paper, in person. The remaining surveys were completed online. Among persons who provided information on age, 50 (12%) survey respondents were 18-30 years of age, 90 (21%) were 31-45, 163 (38%) were 46-60, and 121 (29%) were above 60 years of age. Across all congregations, 46.1% (200/434; 95% CI 41.3-50.9%) reported that they thought they had COVID-19, compared with 39.4% (171/434; 95% CI 34.7-44.1%) who reported testing positive for COVID-19. Among all participants, 9% (40/434) did not receive the COVID-19 vaccine while 88.7% (385/434) completed their primary vaccine series.

Seroprevalence data collected by our team revealed a seropositivity rate of up to 80% for the area where most participants reside or work. Among a subset of persons providing both seroprevalence and survey data, there was concordance in approximately 62% (8/13) of seropositive cases.

**Conclusion:**

The data suggest infection rates reported by a relatively highly vaccinated group of predominantly Black congregants were below that of the general community. Further analyses will examine reasons for these findings.

**Disclosures:**

**All Authors**: No reported disclosures

